# Development of the *Journal of Yeungnam Medical Science*: transition from a local to an international journal

**DOI:** 10.12701/jyms.2026.43.24

**Published:** 2026-03-19

**Authors:** Eun Il Lee, Min Cheol Chang

**Affiliations:** 1 Medical Library, Yeungnam University College of Medicine, Daegu, Korea; 2 Department of Physical Medicine and Rehabilitation, Yeungnam University College of Medicine, Daegu, Korea

**Keywords:** Editorial policies, Journal management, Open access publishing, Periodicals as topic

## Abstract

The medical journal publishing environment in Korea has changed rapidly since the 2010s owing to the expanded influence of international indexing databases, changes in research performance evaluation systems, and the strengthening of research and publication ethics. Amid these changes, the *Journal of Yeungnam Medical Science* (JYMS) has successfully advanced from a local journal to an international journal through a series of strategic transformations, including the appointment of a Managing Editor in 2011, conversion to an English journal in 2018, introduction of an online manuscript submission and peer review system in 2020, and transition to continuous article publishing in 2025. Consequently, it has been indexed in major international indexing databases, including the Directory of Open Access Journals (DOAJ), PubMed, PubMed Central, Scopus, and Emerging Sources Citation Index (ESCI). Notably, in 2025, international submissions increased substantially, with foreign authors accounting for 79.1% of the total submissions. In addition, citation metrics such as Journal Impact Factor and CiteScore increased. These achievements were supported by the dedicated efforts of the editorial board members, volunteer peer reviewers, and the local and international authors who submitted high-quality manuscripts, together with the establishment of standardized editorial policies and systematic quality control procedures. However, practical challenges remain, including financial constraints, staffing structures, workflow continuity, and difficulties securing peer reviewers. The JYMS case demonstrates that journal internationalization is a long-term process enabled by sustained investment, establishment of professional infrastructure, and organizational support. This suggests that collective academic commitment and stable operational systems are essential for the sustainable development of journals.

## Introduction

The publishing environment of medical journals in Korea has undergone rapid changes since the 2010s. The expanded influence of international indexing databases, changes in the researcher performance evaluation system to emphasize publication records in international journals, and the growing importance of research and publication ethics required structural transformation across all journal operations. In this context, for local journals to survive and develop, it was necessary for them to be indexed in international indexing databases, establish editorial and publication systems consistent with the standards of international journals, and strengthen research and publication ethics. To achieve this, a fundamental review and changes across journal operations, including editorial policies, manuscript structure, and publication format, were essential.

Founded in 1984, the *Journal of Yeungnam Medical Science* (JYMS) is a comprehensive medical journal published by Yeungnam University College of Medicine in Korea that covers all fields of medicine. For a long time after its founding, it remained a local journal, published articles primarily by local researchers, and was mainly read by a local audience. At present, JYMS is indexed in major international indexing databases, including the Directory of Open Access Journals (DOAJ), PubMed, PubMed Central, Scopus, and Emerging Sources Citation Index (ESCI); as of 2025, foreign researchers have submitted 330 manuscripts, accounting for 79.1% of the total submissions in 2025. JYMS can be regarded as a representative case of successfully establishing itself as a journal that is consistent with international standards amid the demands of rapid changes in the journal environment.

Here, we aim to examine the development of JYMS, focusing on its transition from a local to an international journal.

## *Journal of Yeungnam Medical Science*: overview and scope

JYMS was founded in 1984 as a Korean journal. Its aim and scope are to provide new medical information not only to various experts in the field of medicine but also to the general public and to contribute to the advancement of medicine by publishing high-quality, evidence-based articles. JYMS broadly covers research across all fields of medicine, including clinical research, basic medical science, and medical education. As a journal published by a university, JYMS places a strong emphasis on research in the field of medical education, targeting learners at different levels, including residents, fellows, and medical students.

## *Journal of Yeungnam Medical Science*: major milestones

**Fig. 1.**
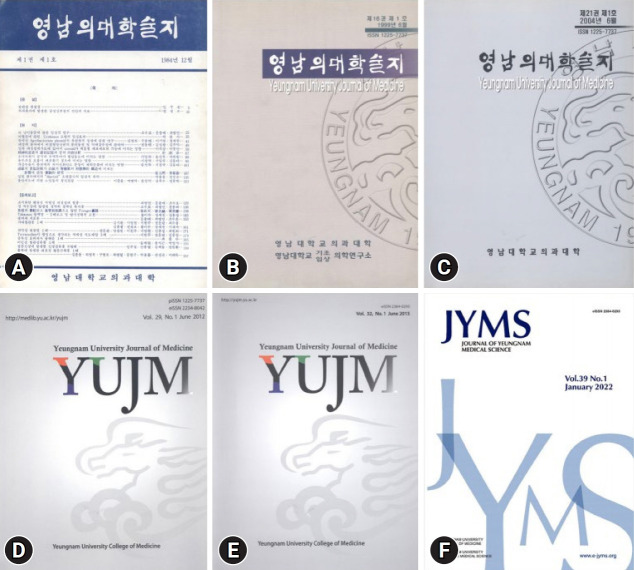
Cover changes of the *Journal of Yeungnam Medical Science*. (A) First issue published in 1984. (B) Volumes 16 (1999) to 20 (2003). (C) Volumes 21 (2004) to 28 (2011). (D) Volumes 29 (2012) to 31 (2014). (E) Volumes 32 (2015) to 38 (2021). (F) New cover beginning with Volume 39 (2022). Reproduced from Park [2] under the Creative Commons Attribution License.

Since its founding in 1984, JYMS has strengthened its foundation as a medical school journal in Korea and has undergone gradual institutional development. At the time of its founding, the journal was titled “영남의대학술지” (Yŏngnam Ŭidae Haksulji). In 1994, it was assigned an International Standard Serial Number (ISSN: 1225-7737) and indexed in KoreaMed in December of the same year, thereby establishing its credibility as a specialized medical journal in Korea. In 2011, following the appointment of the Managing Editor (Eun Il Lee), the editorial and publication systems were reorganized through the standardization of operational regulations and policies. In 2012, the journal format was changed from B5 to US letter size, and the cover and interior layouts were completely redesigned (Fig. 1) [1]. These changes have improved the journal’s external appearance and editorial format to better align it with international standards. In the same year, the English journal title “*Yeungnam University Journal of Medicine* (eISSN: 2234-8042)” was adopted. Notably, in 2015, it was converted into an online-only journal (eISSN: 2384-0293). In 2016, it was selected as a candidate journal in the Korea Citation Index (KCI) by the National Research Foundation (NRF) of Korea; in 2018, it was selected as an accredited journal in the KCI, thereby becoming a recognized medical journal in Korea. Beginning in 2018, it was converted into an English journal, and in 2019, through indexing in PubMed and PubMed Central, it began to develop in earnest as an international journal. Subsequently, in 2022, the journal title was changed from “*Yeungnam University Journal of Medicine*” to “*Journal of Yeungnam Medical Science*,” further clarifying its identity as an international journal rather than one limited to Yeungnam University College of Medicine. In 2023, it was accepted for inclusion in Scopus and ESCI. In 2025, by transitioning from a quarterly publication system to an annual continuous article publishing system, it established a publication structure capable of responding in a more flexible manner to increasing submission demands and international readership. Table 1 summarizes key milestones in the history of JYMS [2]. The annual publication profile and editorial composition from its founding in 1984 to 2025 are presented by manuscript type (Supplementary Table 1). By 2023, the proportion of case reports was relatively high; however, since 2024, the proportion of case reports has decreased, and the number of original articles and review articles has increased. In addition, since 2020, with the introduction of new manuscript types, such as Communications, Imagery, Image Vignette, Resident Fellow Section, and Medical Student Education Section, JYMS has progressively expanded to encompass educational, visual, and short-form scholarly content while maintaining its role as a research-centered journal. These changes demonstrate that the journal has developed in a flexible manner in response to the contemporary demands and needs of diverse readers.

## Efforts and outcomes for journal internationalization

### 1. Conversion to an English journal and international standardization efforts

In 2018, JYMS was fully converted into an English journal. This was a strategic decision to expand the journal’s scope of international readership and submissions. Conversion to an English journal was a minimum prerequisite for international researchers to recognize JYMS as a potential target journal for submission, and it subsequently served as a foundation for indexing in international indexing databases such as DOAJ, PubMed, PubMed Central, Scopus, and ESCI.

For journal internationalization, not only conversion to English but also systematic revisions to the overall editorial standards were undertaken, including manuscript preparation guidelines, reference citation formats, principles for organizing tables and figures, and the overall editorial style. These changes went beyond the levels of conventional Korean journals and were intended to meet the standards and consistencies expected of international journals. Such international standardization was not achieved in a short period but was implemented gradually over several years through sustained effort.

### 2. Establishment of journal website and online manuscript submission and peer review system

The JYMS website has progressively developed as a core infrastructure that determines the journal’s international accessibility and visibility. Until 2014, a website developed internally by the medical library was used, and from 2015 to 2018, accessibility was improved by implementing a mobile web version through an external professional company (XMLink, Seoul, Korea). In 2019, the journal website transitioned to M2PI (Seoul, Korea) to provide improved services. In 2024, the website was completely rebuilt as a responsive website, thereby ensuring stable readability without text distortion across various device environments, including personal computers, tablets, and mobile devices. Such website advancements went beyond simple design improvements and played an important role in enhancing the reliability of scholarly information delivery to international readers and authors.

The manuscript submission and peer review processes also underwent significant changes during the internationalization process of JYMS. Until 2011, a manual system was used, in which manuscripts and review comments were exchanged in paper form. From 2012 to 2016, an email-based submission and peer review system was implemented. Between 2017 and 2019, the Journal Article Management System (JAMS; NRF, Daejeon, Korea), provided free of charge by the NRF, was utilized. However, owing to limitations associated with its Korean-based environment and system stability, there were constraints in fully advancing the journal’s internationalization strategy. Accordingly, in 2020, an online manuscript submission and peer review system provided by M2PI was introduced. This system enabled the stable operation and implementation of diverse functions and served as a foundation for effectively responding to the anticipated increase in overseas submissions following indexing in PubMed, thereby establishing an essential operational infrastructure for JYMS to grow as an international journal.

### 3. Introduction of manuscript editor and qualitative advancement of editorial quality

At JYMS, from 2011 to 2019, the Managing Editor personally performed manuscript editing tasks. In 2020, by introducing professional manuscript editors (Hye-Min Cho, Yoon Joo Seo) from an external institution (InfoLumi, Seoul, Korea), a system was established to review and refine manuscripts at the final publication stage in accordance with the standards of international journals.

The external professional manuscript editors not only corrected grammar and expressions but also comprehensively reviewed the consistency of academic style, logical structure, organization of tables and figures, and compliance with international editorial standards, thereby enhancing the overall manuscript quality. In addition, by providing professional consultation on various practical and technical issues arising in the editorial process, they contributed to decision-making related to the interpretation of editorial standards and international publishing practices.

Such involvement of manuscript editors played an important role not only in improving the quality of individual manuscripts but also in establishing consistency in editorial style across the journal as a whole. Consequently, JYMS came to be recognized as a journal with a unified editorial and publication system, which is considered to have played a key role in meeting the evaluation criteria of major international indexing databases such as Scopus and ESCI.

### 4. Indexing in international databases

**Fig. 2.**
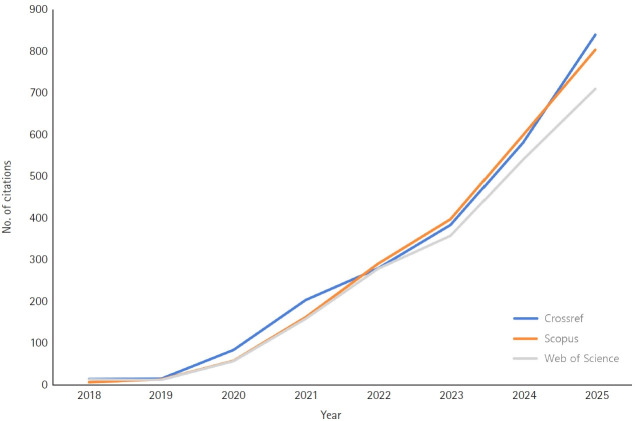
Number of citations of articles published in the *Journal of Yeungnam Medical Science* in Crossref, Scopus, and Web of Science Core Collection from 2018 to 2025 (calculated on December 31, 2025).

JYMS was converted to an English journal in 2018 for inclusion in international indexing databases; its publication frequency increased from biannual to triannual in 2019, and it was indexed in PubMed, PubMed Central, and DOAJ. In 2020, the publication frequency increased from triannual to quarterly. JYMS was then indexed in the Chemical Abstracts Service (CAS), and in 2023, it was indexed in Scopus and ESCI, thereby gaining recognition as an international journal. In addition, the citation metrics in international indexing databases such as Crossref, Scopus, and Web of Science Core Collection have steadily increased (Fig. 2) [3-6].

## Manuscript submission status and changes in international author inflow

**Fig. 3.**
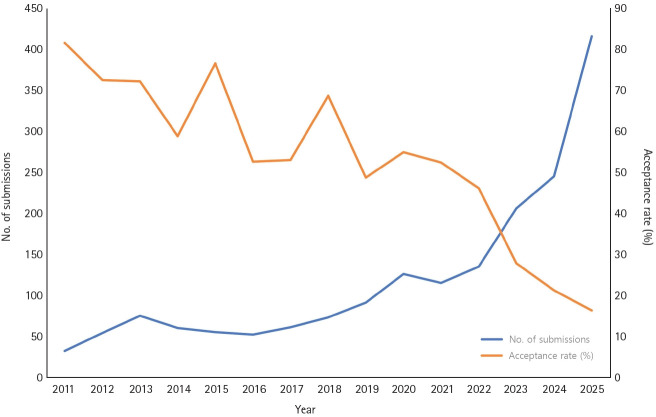
Trends in manuscript submissions and acceptance rate of the *Journal of Yeungnam Medical Science* (2011–2025).

An examination of the annual manuscript submission status of JYMS since 2011 shows a continuously increasing trend. The annual number of submissions, which was 33 in 2011, reached 417 by 2025, representing an increase of more than 10-fold (Fig. 3). This increase can be regarded as the result of the gradual reorganization of the editorial and publication system and its transition toward internationalization. In particular, the conversion to an English journal in 2018 and indexing in PubMed and PubMed Central in 2019 served as important turning points that substantially enhanced the international visibility of JYMS and can be regarded as having significantly contributed to the subsequent increase in submissions. In addition, the acceptance rate continuously decreased from 81.8% in 2011 to 16.9% in 2025, indicating that review standards were strengthened with the increase in submissions (Fig. 3). This decrease in the acceptance rate indicates that the qualitative level of articles published in JYMS is increasing.

In addition, submissions from international authors have steadily increased (Table 2) [6]. Until 2020, submissions to JYMS were primarily from local authors. However, beginning in 2020, submissions from international authors began to increase. As presented in Table 2, the number and proportion of submissions from international authors increased markedly from 13 (10.2%) in 2020 to 330 (79.1%) in 2025. This can be regarded as an important indicator that JYMS has transitioned from a local journal to one recognized by international researchers as a target for submission.

**Fig. 4.**
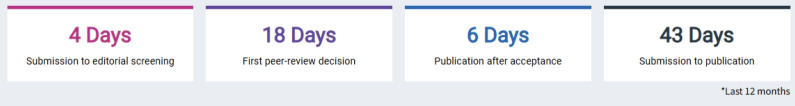
Timeline from manuscript submission to publication in the *Journal of Yeungnam Medical Science* over the last 12 months (https://e-jyms.org; accessed February 10, 2026).

This increase in submissions and the inflow of international authors can be attributed not only to the conversion to an English journal and indexing in international databases, but also to the stable maintenance of a relatively rapid publication system, which functioned as an additional major factor. The average time from manuscript submission to final publication over the past year was approximately 43 days (Fig. 4). Chen et al. [7], in an analysis of health and medical journals indexed in the Science Citation Index Expanded (SCIE), reported that the median submission-to-publication lag was 243.4 days (interquartile range, 159–306 days). Considering this, the publication timeline of JYMS is markedly shorter than the average publication schedule of international medical journals, and this is likely to have functioned as an important factor in providing an environment in which authors can promptly disseminate their research findings.

In addition, JYMS has consistently maintained a policy of not charging an article processing charge (APC) during the submission and publication processes, which is likely to have functioned as an author-friendly factor leading researchers, particularly those from countries with limited research funding support, to recognize JYMS as an attractive venue for submission. Thus, together with securing visibility through inclusion in international indexing databases, the combination of effective editorial and publication management and policy-based approaches can be interpreted as having concurrently expanded the scale of manuscript submissions and the inflow of international authors to JYMS.

## Status of key international database impact indicators for *Journal of Yeungnam Medical Science*

Since being indexed in major international databases such as ESCI and Scopus, JYMS has shown a gradual improvement in impact indicators (Table 3). According to the Web of Science Core Collection, the journal impact factor (JIF) increased from 1.0 in 2023 to 1.4 in 2024, and during the same period, its ranking within the Medicine, General & Internal category improved from 168/325 (51.7%) to 144/332 (43.4%), resulting in a rating ascent from quartile 3 (Q3) to Q2. The Q rankings classify journals within a subject category into four groups based on their impact indicators, with Q1 representing the top 25%, followed by Q2, Q3, and Q4. The Scopus CiteScore showed a similar upward trend from 0.8 in 2023 to 2.0 in 2024, and accordingly, the ranking in the Multidisciplinary category improved from 113/171 (66.1%) to 71/200 (35.5%), also shifting its rating from Q3 to Q2.

Meanwhile, the 2025 impact indicators are scheduled for official release in June 2026. Based on the citation trends to date, the JIF is projected to be approximately 2.0, which is expected to maintain the Q2 status. The CiteScore is projected to increase to approximately 3.1 (https://www.scopus.com/sourceid/21101189035; accessed March 19, 2026). Such projections suggest that the standing of JYMS as an international journal will continue to rise.

## Challenges and future tasks of the *Journal of Yeungnam Medical Science*

While growing into an international journal, JYMS has encountered numerous practical challenges.

First, financial constraints are the most significant practical difficulty in advancing journal operations. Core infrastructure such as establishment of the journal website and online manuscript submission and peer review system, and the introduction of professional manuscript editors is essential for journal internationalization; however, securing the necessary budget for these initiatives was by no means easy. This suggests that journal internationalization requires sustained investment, internal consensus, and support within the organization.

Second, the limitations in the financial structure associated with maintaining an APC-free policy remain an important challenge. JYMS has maintained a policy of not charging an APC, which has had a positive impact on the inflow of international authors and an increase in submissions. However, the journal budget was more than two-fold higher in 2025 than in 2011, and with the expansion in the number of published articles, the financial burden of editorials and publication costs has also continued to grow. Since 2024, partial financial support has been received through the Journal Internationalization Support Program of the Korean Federation of Science and Technology Societies (KOFST); however, the sustainability of external funding remains uncertain. The introduction of an APC may represent a possible alternative; however, as it may lead to a decrease in submissions or article quality, careful consideration is required, and seeking a balance between journal growth and financial stability is necessary.

Third, difficulties in securing peer reviewers intensified with an increase in manuscript submissions. JYMS maintains the principle of review by at least two expert reviewers; however, difficulties in obtaining reviewer acceptance have led to delays in peer review and an increased operational burden. Expansion of the section editor system has been discussed as a measure to address this issue; however, given the comprehensive scope of the journal, the number of currently secured personnel remains limited, and the recruitment of additional editors is not an easy task.

JYMS has set inclusion in SCIE as a mid- to long-term goal, and its overall development direction can be evaluated as consistent with international standards; however, managing the anticipated increase in manuscript submissions, peer review workload, and related administrative tasks accompanying future improvements in performance indicators remains an important task for JYMS.

## Conclusion

Since the 2010s, amid changes in the scholarly publishing environment, JYMS has advanced from a local journal to an international journal through its conversion to an English publication, establishment of an online manuscript submission and peer review system, adoption of internationally standardized editorial practices, and inclusion in major international indexing databases. In particular, since 2011, consistency in editorial policies and operational systems has been maintained, enabling practical advancement of the internationalization strategy, which has served as the foundation for inclusion in major databases such as DOAJ, PubMed, PubMed Central, Scopus, and ESCI. In addition, the combination of a rapid publication system and an APC-free policy led to a substantial increase in submissions from international authors, and the journal’s international standing and influence improved.

However, despite these achievements, challenges such as financial constraints, issues related to staffing structure and continuity of work, and difficulties in securing peer reviewers remain. Internationalization requires long-term investment and internal organizational support, and for sustainable operations in the future, it is necessary to have financial stability, expand professional personnel, and strengthen the operational system. The experience of JYMS suggests that journal internationalization cannot be achieved through short-term outcomes alone but requires concurrent infrastructure development, enhancement of personnel expertise, and long-term investment.

## Figures and Tables

**Table 1.** The history of the development of the *Journal of Yeungnam Medical Science*

Date	Key milestones
Dec 1984	The journal was launched in 1984 as a Korean journal titled *Yŏngnam Ŭidae Haksulji*
Jul 1994	International standard serial number was assigned (pISSN: 1225-7737)
Dec 2004	The journal was indexed in KoreaMed
Apr 2011	Appointment of a Managing Editor
Jun 2012	The journal’s English name was added as *Yeungnam University Journal of Medicine* (eISSN: 2234-8042)
	Comprehensive redesign of journal format, cover, and layout (transition from B5 size to US letter size)
Feb 2013	The journal was indexed in KoreaMed Synapse, and digital object identifiers (DOIs) were assigned
Jun 2015	The journal began publishing articles online only
	The journal name remained the same (*Yeungnam University Journal of Medicine*, eISSN: 2384-0293)
	Launch of a new journal website by XMLink (Seoul, Korea)
Dec 2016	The journal was indexed as a candidate journal in the Korea Citation Index (KCI)
Jun 2018	The journal adopted an English-only policy and online manuscript submission and peer review systems were introduced (Journal Article Management System [JAMS]; National Research Foundation [NRF], Daejeon, Korea)
Oct 2018	The journal was indexed as an accredited journal in KCI
Jan 2019	Increase in publication frequency from biannual to triannual
Jun 2019	The journal was indexed in the Directory of Open Access Journals (DOAJ)
Oct 2019	The journal was indexed in PubMed Central (PMC) and became searchable through PubMed
Jan 2020	International-level manuscript editing was implemented through professional manuscript editors (InfoLumi, Seoul, Korea)
	Increase in publication frequency from triannual to quarterly
	Launch of a new journal website and online manuscript submission and peer review system (M2PI, Seoul, Korea)
Oct 2020	The journal was indexed in Chemical Abstracts Service (CAS)
	The journal expanded the types of articles published with the launch of Communications.
Jan 2022	The journal’s name was changed to Journal of *Yeungnam Medical Science* (eISSN: 2799-8010)
	The journal expanded the types of articles published with the launch of Imagery, Image Vignette, and Resident Fellow sections.
Mar 2023	The journal was accepted for inclusion in Scopus
Sep 2023	The journal was accepted for inclusion in Emerging Sources Citation Index (ESCI)
Jan 2024	Launch of a responsive website for multi-device compatibility
Jan 2025	Transition from quarterly publication to continuous article publishing with annual compilation
Nov 2025	The journal expanded the types of articles published: Medical Student Education Section was launched

Adapted from Park [2] under Creative Commons Attribution License.

**Table 2.** Trends in manuscript submissions to the *Journal of Yeungnam Medical Science* by author origin (2020–2025)

Year	Local	International	Total
2020	114 (89.8)	13 (10.2)	127
2021	107 (92.2)	9 (7.8)	116
2022	81 (59.6)	55 (40.4)	136
2023	99 (47.8)	108 (52.2)	207
2024	72 (29.3)	174 (70.7)	246
2025	87 (20.9)	330 (79.1)	417

Data are presented as number (%). Adapted from Kim [6] under Creative Commons Attribution License.

**Table 3.** Journal impact factor and CiteScore of the *Journal of Yeungnam Medical Science* (2023–2025)

	2023	2024	2025	Category
Journal impact factor^a)^	1.0	1.4	2.0^b)^	Medicine, general & internal
Rank	168/325 (51.7%)	144/332 (43.4%)		
Rating	Q3	Q2		
CiteScore	0.8	2.0	3.1^c)^	Multidisciplinary
Rank	113/171 (66.1%)	71/200 (35.5%)		
Rating	Q3	Q2		

Q, quartile.

a) Journal Citation Reports (Clarivate 2024, 2025).

b) Data expected in June 2026 (calculated March 10, 2026).

c) Scopus CiteScore tracker 2025. Data is updated monthly (accessed March 10, 2026, https://www.scopus.com/sourceid/21101189035).

## Supplementary material

Supplementary Table 1 can be found at https://doi.org/10.12701/jyms.2026.43.24.

Supplementary Table 1. Annual publication profile and editorial composition of *Journal of Yeungnam Medical Science* by article type (1984–2025)
