# Supplementary material for: Development of the Journal of Yeungnam Medical Science: transition from a local to an international journal
Source: J Yeungnam Med Sci. 2026 Mar 19;43:24. doi: 10.12701/jyms.2026.43.24 (PMC13107086; doi:10.12701/jyms.2026.43.24)
Supplement: Supplementary Table 1. — Annual publication profile and editorial composition of Journal of Yeungnam Medical Science by article type (1984–2025) [file jyms-2026-43-24-Supplementary-Table-1.pdf]

Supplementary Table 1. Annual publication profile and editorial composition of *Journal of Yeungnam Medical Science* by article type (1984–2025)

	Volume	Editorial	Review article	Original article	Case report	Communications	Image vignette	RFS section	Medical student education section	Total	Editor-in-Chief	No. of editorial board members
1984	1		2	12	12					26	Pock Soo Kang	4
1985	2		2	27	11					40	Wun Yong Chung	4
1986	3		3	34	15					52		
1987	4		3	29	17					49	Joo Chul Ihn	4
1988	5		4	30	23					57		
1989	6		3	38	12					53		
1990	7		4	29	16					49	Sung-Deok Jung	5
1991	8		4	36	13					53	Bong Sub Shim	5
1992	9		3	33	9					45	Ki Hong Kim	5
1993	10		4	37	12					53	Sung Sae Han	7
1994	11		4	35	7					46		
1995	12		4	25	6					35	Young Soo Huh	8
1996	13		6	23	6					35		
1997	14		8	27	10					45	Kwang Youn Lee	8
1998	15		6	26	6					38		
1999	16		6	31	9					46	Han Ku Moon	8
2000	17		4	11	5					20		
2001	18		4	22	3					29	Sei-One Shin	8
2002	19		4	8	4					16		
2003	20		4	13	9					26	Dong Chul Lee	11
2004	21		4	15	12					31		
2005	22		4	14	9					27	Jin Hong Chung	11
2006	23		4	13	16					33		
2007	24		32	62	37					131		
2008	25		3	5	18					26		
2009	26		4	3	16					23	Jae Ho Cho	11
2010	27		4	4	19					27		
2011	28		4	7	16					27	Joon Sakong	13
2012	29		4	3	26					33		
2013	30		3	4	26					33	Hosun Park	13
2014	31		1	5	26					32		
2015	32		1	4	27					32		
2016	33		2	6	28					36		
2017	34		7	14	29					50	Joon Hyuk Choi	24
2018	35		8	14	19					41		
2019	36		10	17	14					41		
2020	37	3	15	17	19	1				55		
2021	38	1	15	16	23					55	So-Young Park	49
2022	39	2	10	22	17	1	1	1		54		
2023	40	4	14	35	29	4	3	6		95		
2024	41	2	15	14	8	2	1	1		43		
2025	42	3	17	35	11	3	9	1	1	80	Tae Gon Kim	44
Total		15	263	855	650	11	14	9	1	1,818		

RFS, Resident Fellow Section.
